# Arthroscopic High‐in‐the‐Groove Biceps Tenodesis: Loop‐and‐Tack Technique

**DOI:** 10.1002/atn2.70105

**Published:** 2026-07-13

**Authors:** Marc Nicholes, Micah Ngatuvai, Nata Parnes, John P. Scanaliato

**Affiliations:** ^1^ Department of Orthopaedic Surgery William Beaumont Army Medical Center Ft Bliss Texas U.S.A.; ^2^ Department of Orthopaedic Surgery Texas Tech University Health Sciences Center El Paso Texas U.S.A.; ^3^ Department of Orthopaedic Surgery Carthage Area Hospital Carthage New York U.S.A.; ^4^ Department of Orthopaedic Surgery Claxton‐Hepburn Medical Center Ogdensburg New York U.S.A.

## Abstract

Arthroscopic biceps tenodesis is an effective treatment for patients with long head of the biceps tendon pathology. This technical note details a high‐in‐the‐groove arthroscopic technique utilizing loop‐and‐tack fixation through a single cannula. This technique utilizes a spinal needle to create the “tack” portion of the construct, minimizing the risk of iatrogenic injury associated with the use of a BirdBeak or other sharp penetrating instrument. In addition, the biceps anchor remains intact during much of the technique, which allows for easier preservation of long head of the biceps tendon tension. Finally, it minimizes the use of additional cannulas and/or one‐time use instruments, which may decrease the overall cost compared with other techniques.

## Graphical abstract

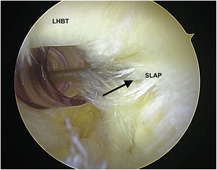

**VIDEO 1:** Performance of a high‐in‐the‐groove arthroscopic biceps tenodesis. Glenohumeral view of the left shoulder from the posterior viewing portal. Video content can be viewed at https://doi.org/10.1002/atn2.70105.

Pathology of the long head of the biceps tendon (LHBT) is a common source of anterior shoulder pain, frequently occurring alongside rotator cuff tears and SLAP lesions.^1^ When conservative management fails, biceps tenodesis provides excellent functional outcomes while also preserving cosmetic appearance and biceps muscle function while minimizing the risk of postoperative cramping.

Open subpectoral biceps tenodesis has often been considered the gold standard technique for the performance of biceps tenodesis. However, open techniques require larger incisions that are often bothersome for patients due to their location. There is also a risk, albeit low, of injury to neurovascular structures through this approach.^2^, ^3^

Arthroscopic biceps tenodesis has been shown to have equivalent outcomes to open techniques.^4^, ^5^ Methods originally detailed by Farmer inspired our technique, but we believe our approach has further optimized the procedure, making it ideal for residents and less‐experienced arthroscopists alike.^6^

## SURGICAL TECHNIQUE

### Setup and Portal Placement

**FIGURE 1:**
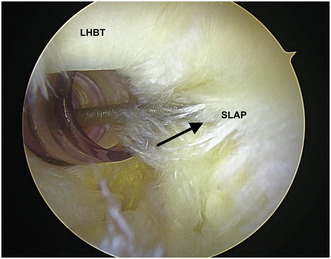
Diagnostic arthroscopy showing fraying and instability of the superior labrum (arrow). Glenohumeral view of the left shoulder from the posterior viewing portal. (LHBT, long head of the biceps tendon; SLAP, superior labrum from anterior to posterior tear.)

This technique can be performed with the patient in both the beach chair and lateral decubitus positions. A posterior portal is established based on the surgeon's preference. The arthroscope is then introduced into the glenohumeral joint, and the superior labrum and biceps undergo a cursory evaluation (Video 1). An anterior portal is then localized with an outside‐in technique and cannulated with a 7 × 7 cm theaded‐in cannula (Arthrex, Naples, FL). If preoperative symptoms or cursory evaluation suggest injury to the superior labrum and/or LHBT, and we suspect subsequent performance of an arthroscopic tenodesis, this portal is placed superior and lateral within the rotator interval, just inferior to the biceps tendon and superior to the subscapularis tendon. A comprehensive diagnostic arthroscopy is performed in the standard fashion (Figure 1).

### Tenodesis Site Preparation and Luggage‐Tag Creation

**FIGURE 2:**
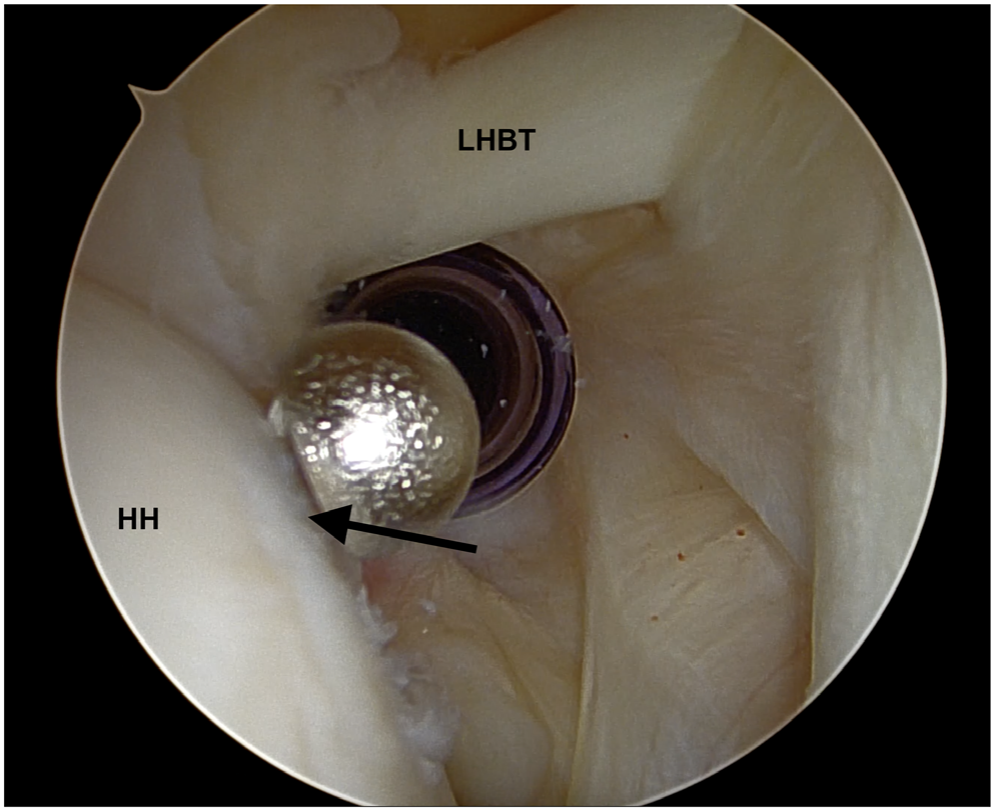
The humeral cartilage (arrow) immediately superior to the subscapularis tendon, just proximal to the biceps pulley is gently decorticated with a mechanical shaver. Glenohumeral view of the left shoulder from the posterior viewing portal. (HH, humeral head; LHBT, long head of the biceps tendon.)

**FIGURE 3:**
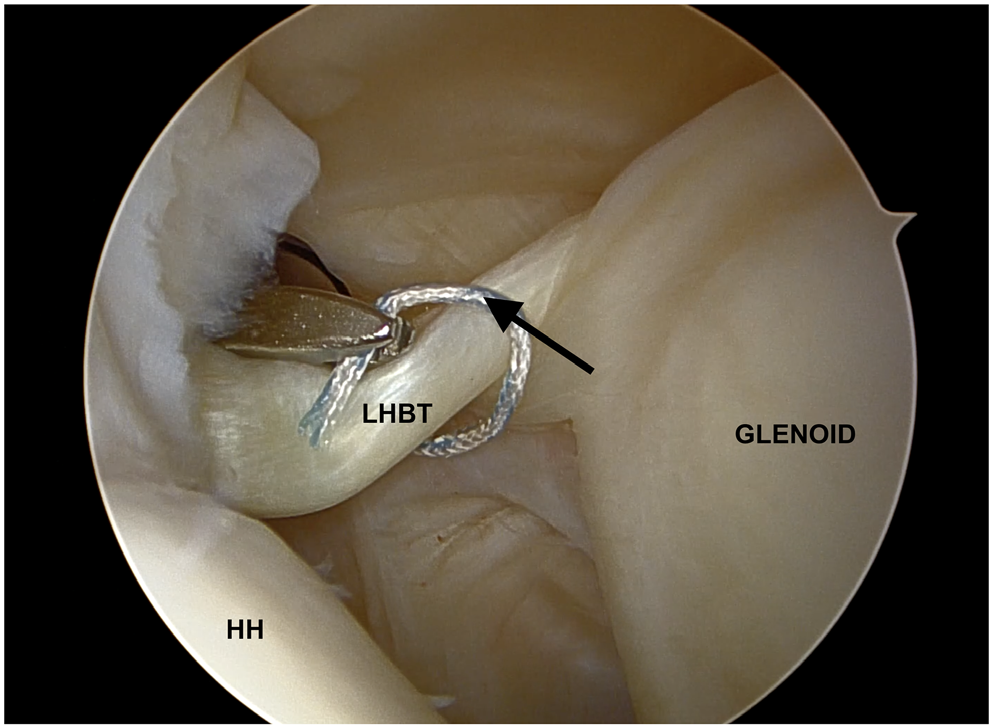
The free end of the FiberLink suture (arrow) is passed around the LHBT from inferior to superior. Glenohumeral view of the left shoulder from the posterior viewing portal. (HH, humeral head; LHBT, long head of the biceps tendon.)

**FIGURE 4:**
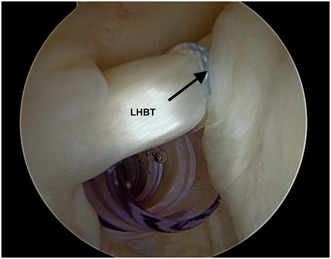
The luggage tag is created and shuttled as medial as possible (arrow). Glenohumeral view of the left shoulder from the posterior viewing portal. (LHBT, long head of the biceps tendon.)

Once the decision to perform arthroscopic tenodesis is made, the planned tenodesis site is gently decorticated and denuded of nonarticulating cartilage with an arthroscopic shaver. We perform the tenodesis immediately superior to the subscapularis and immediately inferomedial to the biceps pulley (Figure 2). A KingFisher grasper (Arthrex) introduced through the anterior cannula passes the free end of a FiberLink suture (Arthrex) into the glenohumeral joint on the inferior aspect of the biceps tendon (Figure 3). Using the same grasper, the suture is retrieved on the superior aspect of the tendon. Outside of the cannula, the free end of the suture is passed through the looped end of the suture and tensioned, creating a “luggage tag.” It is important to aim the cannula medially during this step, to medialize the luggage tag as close to the biceps anchor as possible (Figure 4).

### Percutaneous Needle and Shuttle Suture Passage

**FIGURE 5:**
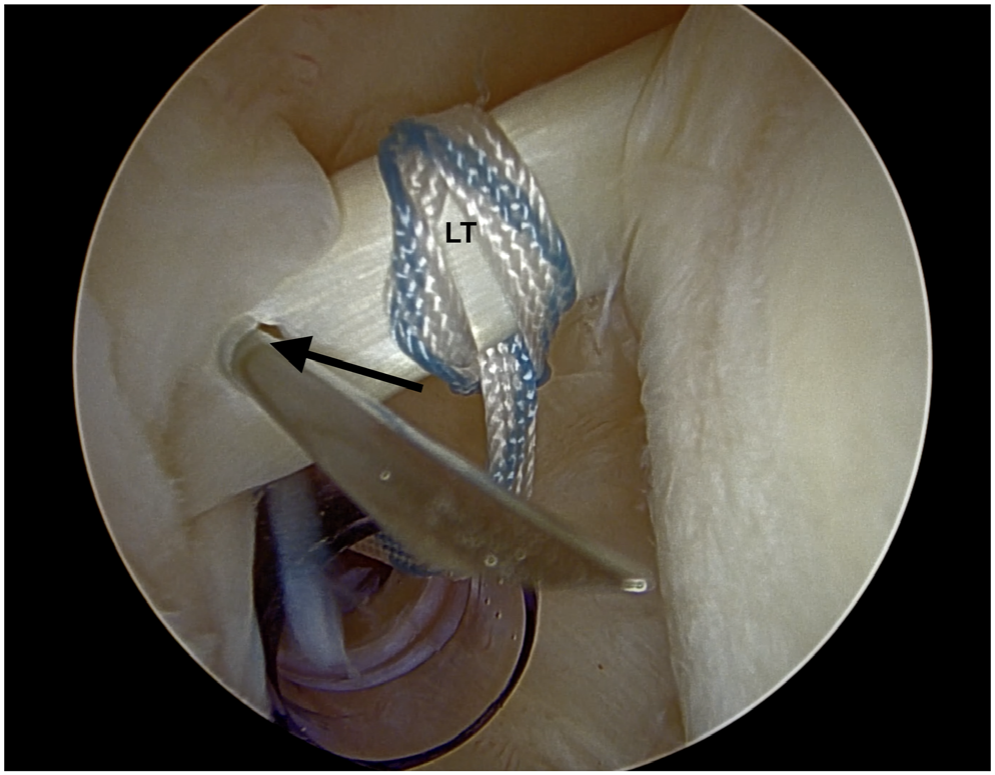
A percutaneously placed spinal needle is used to penetrate the LHBT distal to the luggage tag (arrow). Glenohumeral view of the left shoulder from the posterior viewing portal. (LHBT, long head of the biceps tendon; LT, luggage tag.)

**FIGURE 6:**
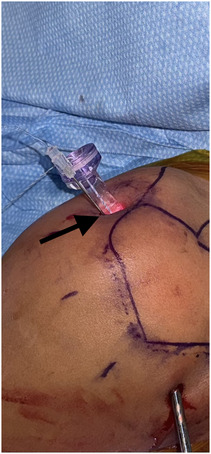
Left shoulder in the beach chair position. The entry location for the spinal needle is typically just superior and lateral to the anterior cannula (arrow).

**FIGURE 7:**
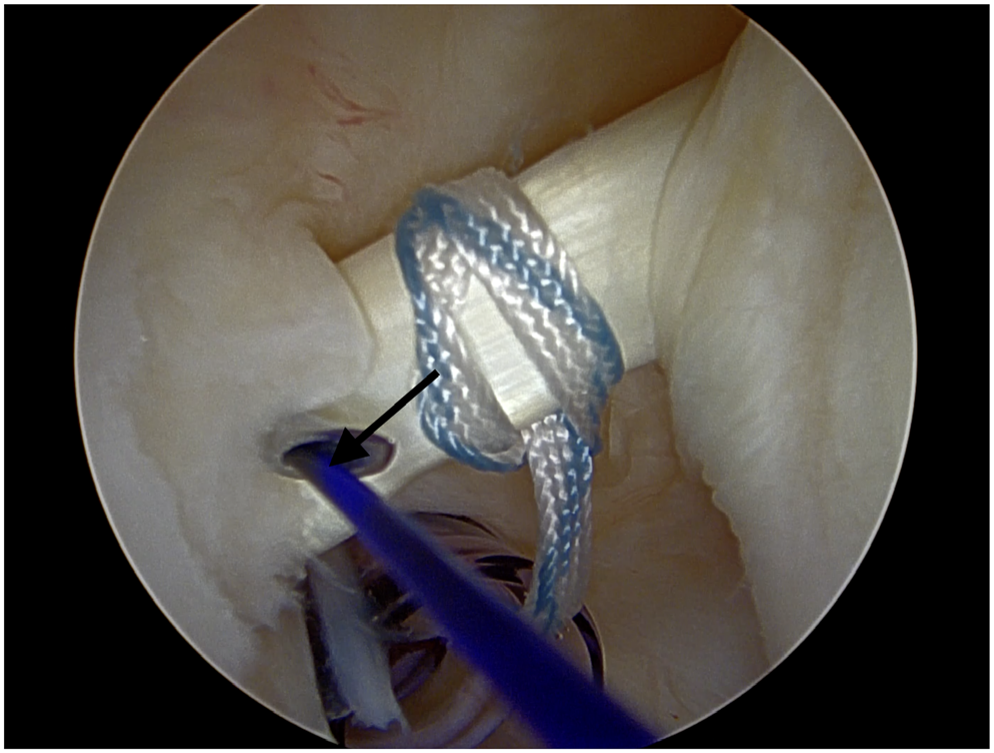
A PDS suture (arrow) is passed through the spinal needle for tack creation. Glenohumeral view of the left shoulder from the posterior viewing portal. (PDS, polydioxanone.)

**FIGURE 8:**
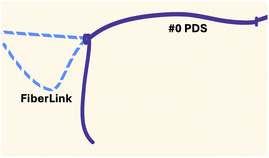
Graphical representation of the PDS/FiberLink construct. The knot without the FiberLink is used to dilate the tendon for easier passage of the PDS/FiberLink. (PDS, polydioxanone.)

**FIGURE 9:**
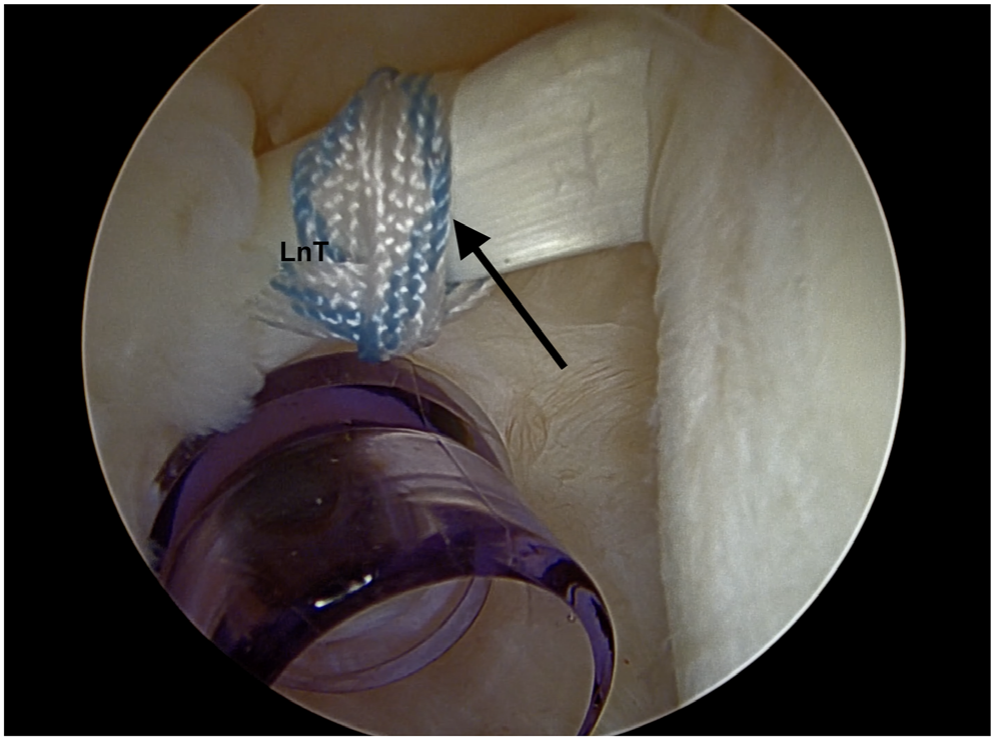
The PDS is withdrawn, shuttling the FiberLink percutaneously and creating the tack of the construct. It is important to direct the loop laterally (arrow) to allow for subsequent tenotomy with preservation of 6 to 8 mm of LHBT stump. Glenohumeral view of the left shoulder from the posterior viewing portal. (LHBT, long head of the biceps tendon; LnT, loop and tack; PDS, polydioxanone.)

**FIGURE 10:**
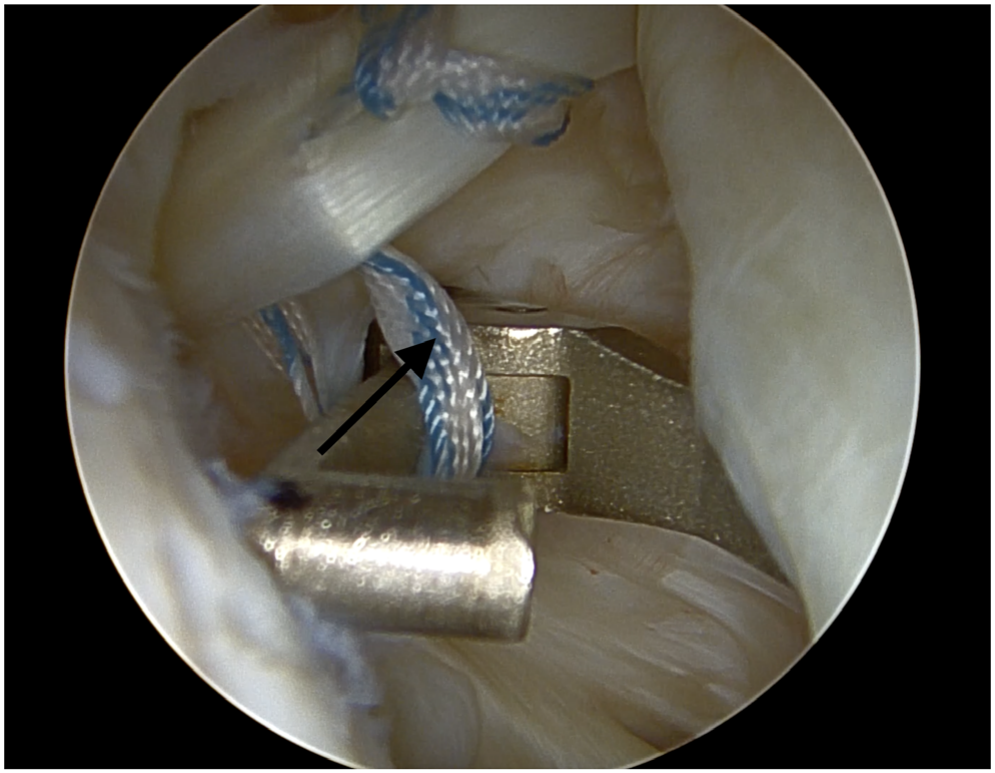
The FiberLink suture is retrieved through the anterior cannula with a Tape Grasper (arrow). Glenohumeral view of the left shoulder from the posterior viewing portal.

To create the tack component of the fixation, an 18‐gauge spinal needle is percutaneously placed through the biceps tendon, lateral to the luggage tag loop, as close as possible to the planned tenodesis site (Figure 5). Typically, the percutaneous entry site is immediately superior and lateral to the anterior cannula (Figure 6). The stylet is removed from the spinal needle, and a 2‐0 polydioxanone (PDS) suture (Ethicon, Raritan, NJ) is passed through the needle into the joint (Figure 7). The PDS suture is retrieved with a KingFisher grasper (Arthrex) out of the anterior cannula. Exterior to the cannula, approximately 10 cm distal to the retrieved end of the PDS suture, a single overhand knot is tied. A second overhand knot is tied approximately midway between the end of the PDS suture and the first knot, and the free end of the FiberLink suture is loaded into this knot for subsequent shuttling (Figure 8). The first knot serves to dilate the tendon slightly, allowing for easier passage of the second knot. The spinal needle is now completely withdrawn, and the percutaneous PDS is pulled, shuttling the FiberLink through the tendon lateral to the loop, thereby creating the “tack” component of the construct (Figure 9). A Tape Grasper (Arthrex) is introduced through the anterior cannula, and the FiberLink suture is retrieved through the anterior cannula (Figure 10).

### Tenotomy and Anchor Fixation

**FIGURE 11:**
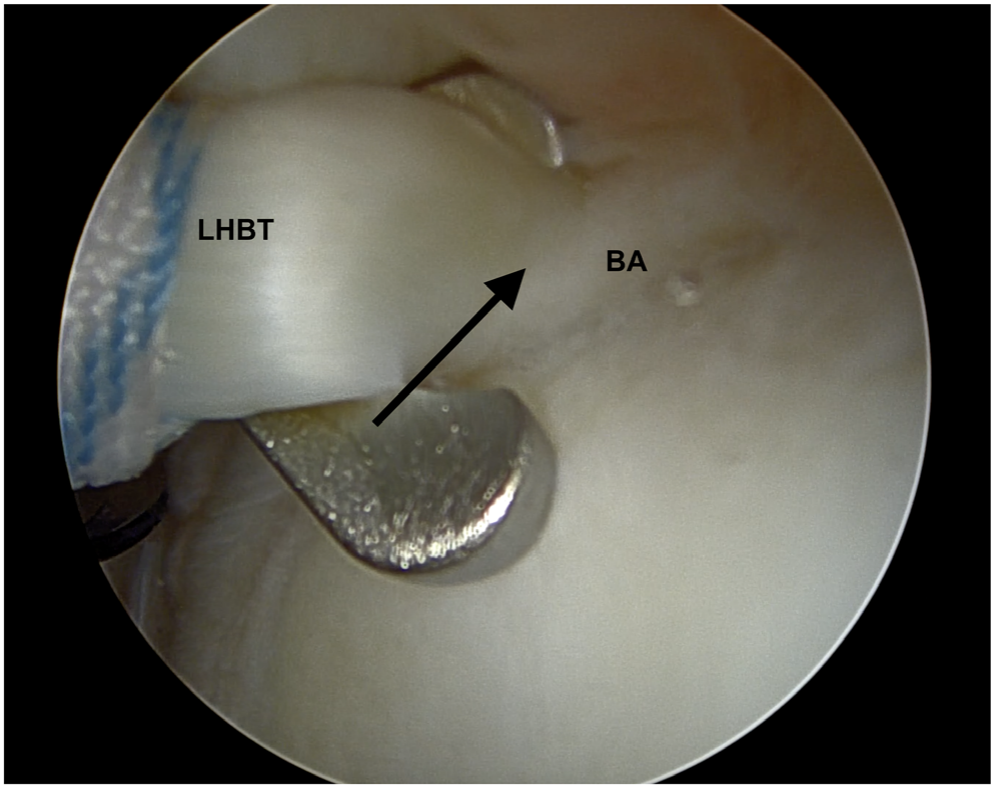
The LHBT is tenotomized just distal to its insertion at the superior labrum (arrow). Glenohumeral view of the left shoulder from the posterior viewing portal. (BA, biceps anchor; LHBT, long head of the biceps tendon.)

**FIGURE 12:**
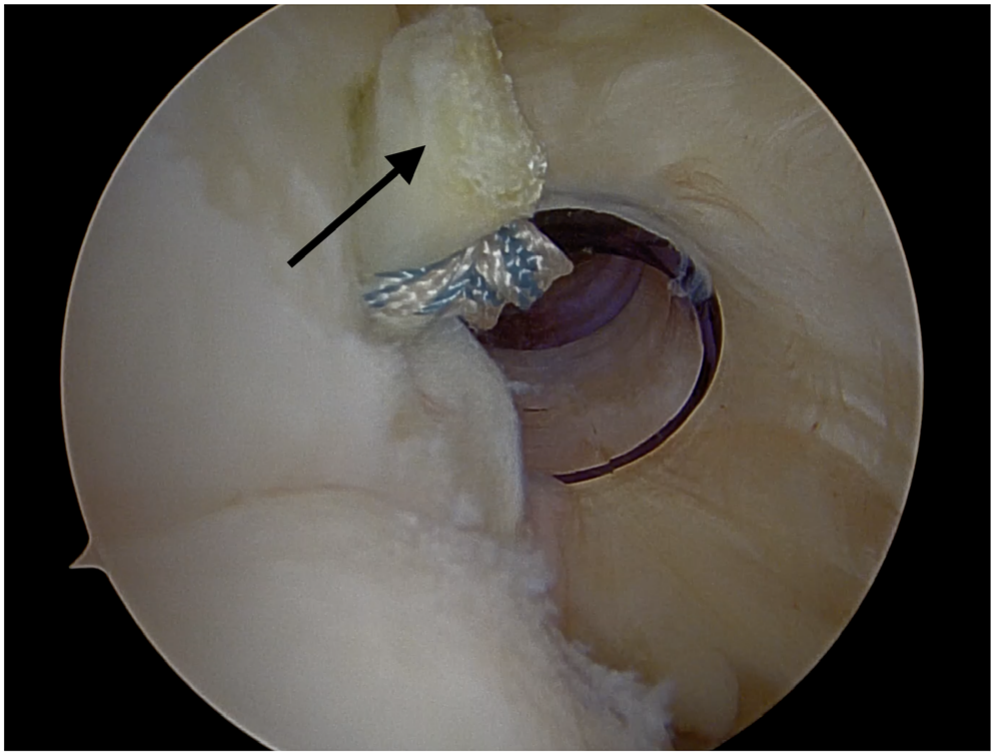
Tenotomy is performed such that 6 to 8 mm of stump is preserved distal to the suture loop (arrow). Glenohumeral view of the left shoulder from the posterior viewing portal.

**FIGURE 13:**
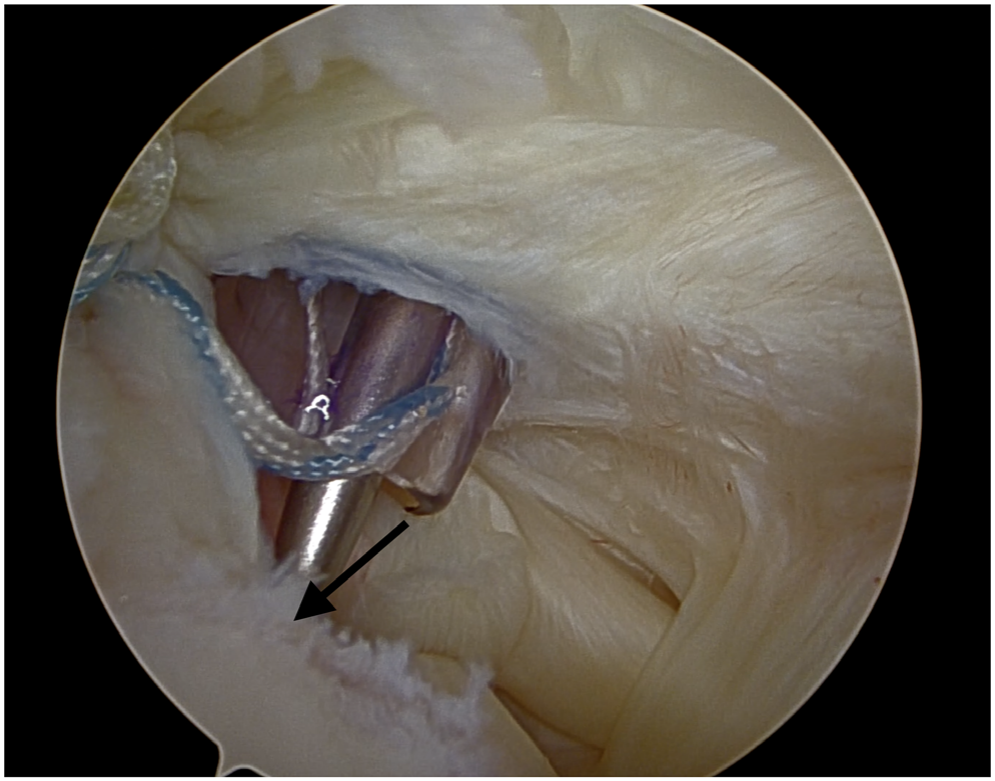
The pilot hole for the anchor is punched just distal to the prepared proximal humeral site (arrow). Glenohumeral view of the left shoulder from the posterior viewing portal.

**FIGURE 14:**
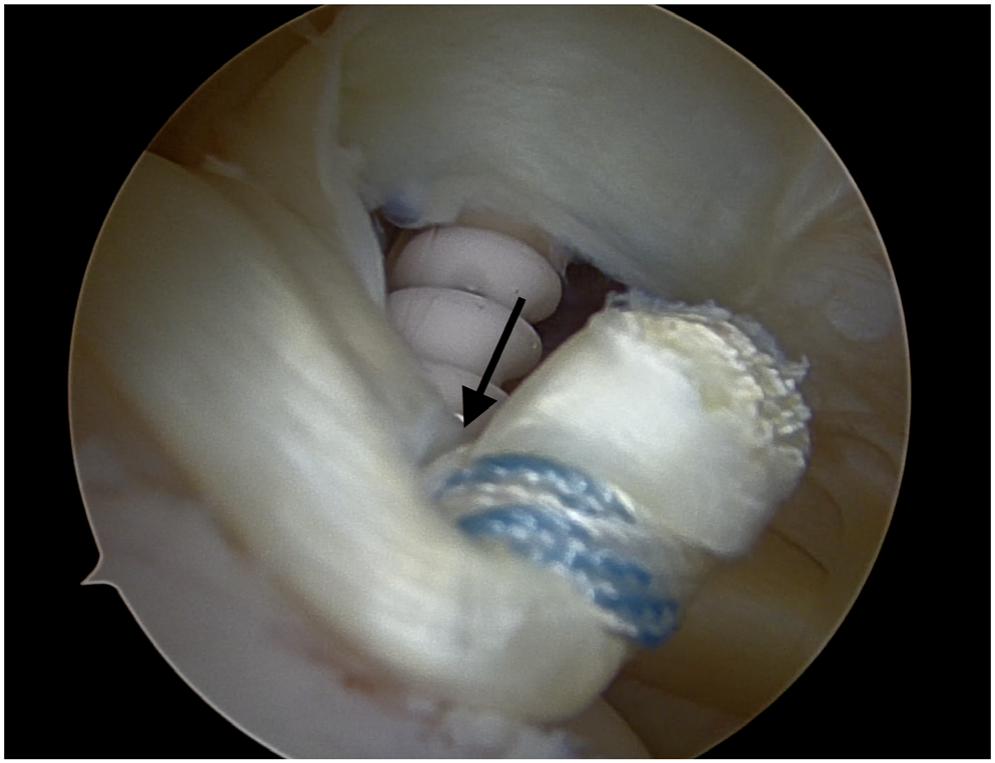
The anchor is loaded with the free end of the suture, and the anchor is advanced (arrow) and LHBT tensioned in the standard fashion. Glenohumeral view of the left shoulder from the posterior viewing portal. (LHBT, long head of the biceps tendon.)

**FIGURE 15:**
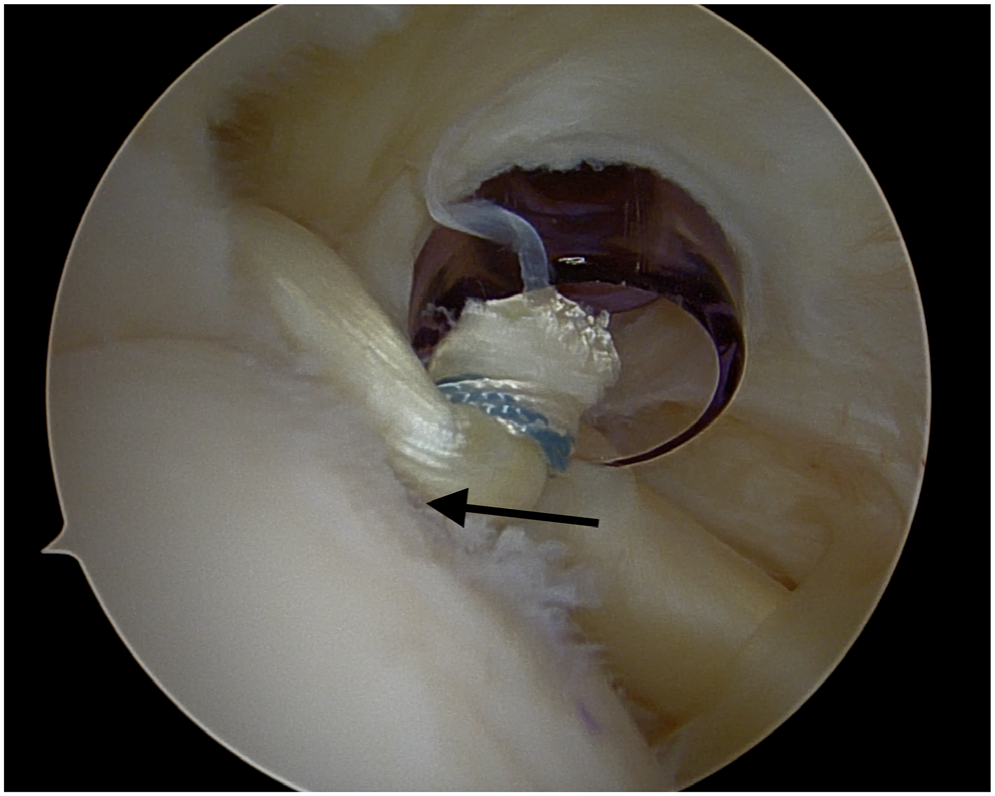
Final assessment of LHBT tenodesis which shows abutment of residual tendon to the area of prepared proximal humerus (arrow). Glenohumeral view of the left shoulder from the posterior viewing portal. (LHBT, long head of the biceps tendon.)

Tenotomy of the LHBT is now performed, either with arthroscopic scissors (Arthrex) or an arthroscopic ablator (Figure 11). It is important to make the tenotomy as medial as possible, to allow for approximately 6 to 8 mm of biceps to remain medial to the loop‐and‐tack construct (Figure 12). The arm is then brought into approximately 10° of forward elevation and slight external rotation. The free end of the FiberLink is loaded into the 3.9 mm SwiveLock anchor (Arthrex), and a pilot hole is punched at the distal‐most aspect of the prepared tenodesis site (Figure 13). The punch is withdrawn, and the anchor is advanced in a collinear fashion into the pilot hole until the anchor body approximates the aperture of the pilot hole (Figure 14). The free end of the suture is pulled until the biceps abuts the prepared tenodesis site, and the anchor is placed in the standard fashion. The suture tail is cut with a closed suture cutter (Arthrex) and the final tenodesis is assessed (Figure 15).

### Postoperative Rehabilitation Protocol

If concomitant rotator cuff or labral repair is performed, those respective protocols take precedence. For isolated tenodesis, the patient is instructed to wear a simple shoulder sling “full time” for the first 2 weeks. Pendulum exercises can begin once the preoperative nerve block has worn off. Following the 2‐week postoperative visit, the sling can be removed in controlled settings, but should be worn in public or while sleeping. Resisted shoulder abduction and flexion, elbow flexion, and forearm supination are restricted for the first 4 weeks, and weight‐bearing is restricted to 5 lbs through the operative shoulder for the first 6 weeks. Gradual strengthening is begun at the 6‐week postoperative visit, and restrictions are removed at the 3‐month postoperative visit.

## DISCUSSION

Choosing between the various LHBT tenodesis options available can be daunting. Surgeons have various approaches, tenodesis locations, tendon positions, and fixation constructs to choose from.^7^ The decision on which technique to utilize is only obfuscated following review of contemporary literature, which suggests no clear superiority of one specific technique.^7^ We believe that this technique offers a simple, effective, and reproducible approach to treat LHBT and/or superior labral pathology and affords surgeons a reliable arthroscopic option to offer patients during a preoperative shared‐decision‐making session (Table 1).

**TABLE 1:** Pearls and Pitfalls

Pearls	Pitfalls
Superolateral placement of portal within interval for ease of anchor placement	If anterior cannula is placed centrally within the interval, placement of the SwiveLock is difficult
Pass free end of FiberLink for easier suture management	Passing the looped end may lead to inadvertent knot formation
Tenotomy prior to anchor placement to prevent over‐tensioning of tendon	Tension with an intact LHBT anchor may lead to anchor eyelet‐body dissociation
Tape grasper allows for less resistance when retrieving percutaneous suture through cannula	A luggage tag placed too laterally may lead to difficulty with tack creation
Anterior cannula angled medially helps achieve ideal position for luggage tag	Tendon penetration with a BirdBeak or other sharp instrument may cause iatrogenic injury to the humeral and/or glenoid cartilage
A simple knot in the PDS helps dilate for the subsequent suture passage through the tendon	

LHBT, long head of the biceps tendon.

A network meta‐analysis by Hurley et al. examined various surgical options for biceps tendon pathology and found no significant differences in multiple outcome measures when comparing open vs arthroscopic biceps tenodesis.^8^ Similarly, a systematic review by Abraham et al. investigating arthroscopic versus open biceps tenodesis found no significant difference in Constant, American Shoulder and Elbow Surgeons or Single Assessment Numeric Evaluation scores between the 2 approaches.^9^ Dekker et al. evaluated the biomechanical properties of various biceps tenodesis techniques and found no significant differences in construct strength when comparing suprapectoral versus subpectoral fixation locations or between different fixation types, including interference screws, suture anchors, and cortical buttons.^10^ Interestingly, contemporary all‐suture anchors appear not only equivalent, but superior to more classical metal anchors with respect to fixation strength, highlighting the biomechanical strength of newer anchor technology.^11^ It warrants mentioning, however, that there is data suggesting that greater tendon migration correlates with lower patient‐reported outcome scores, highlighting the importance of sound surgical technique.^12^ Taken altogether, these biomechanical and clinical findings suggest that the individual surgeon's technical proficiency and achievement of secure fixation are more critical determinants of success than the specific method or location chosen.

Despite the overall equivalence in outcomes, specific differences between arthroscopic and open approaches warrant discussion. Open subpectoral tenodesis may provide more reliable relief of bicipital groove pain, as it allows for complete and direct inspection and treatment of groove pathology. Conversely, arthroscopic techniques offer superior cosmesis with smaller incisions and are associated with fewer wound‐related complications.^9^ In our experience, patients with predominantly LHBT pathology, significant tendinosis, or persistent groove tenderness may benefit from an open subpectoral approach, which addresses the entire pathologic segment of the tendon. In contrast, patients with primary SLAP pathology or those with concerns regarding cosmetic appearance may be better served by an arthroscopic proximal tenodesis technique.

Although multiple techniques for arthroscopic tenodesis have been described, we believe ours offers distinct advantages compared with other arthroscopic techniques (Table 2). The use of a spinal needle to generate the tack minimizes the risk of both eccentric passage through the tendon and iatrogenic injury to the glenohumeral cartilage. Establishing the loop‐and‐tack suture configuration with an intact tendon allows for near‐anatomic restoration of LHBT tension. Performing the tenodesis itself following tenotomy reduces the risk of implant‐related complications. No additional portals are required, which can reduce operative time, minimize cost, and improve postoperative cosmesis. Important for surgeons who train residents, this technique, in our opinion, affords a faster learning curve compared with other arthroscopic techniques.

**TABLE 2:** Advantages and Disadvantages

Advantages	Disadvantages
Spinal needle is more precise and less traumatic than BirdBeak passer	Unable to assess split thickness tears or other distal tendon pathology
Prevents overtensioning of the biceps tendon	Less robust fixation compared with other techniques
Can be done with only 2 portals if no rotator cuff or labral pathology is being addressed	Studies suggest potential for increased stiffness compared with open approach
Improved cosmesis compared with open approach	Difficult to perform concomitant arthroscopic distal clavicle excision if anterior portal is in ideal position for the tenodesis
Faster than open tenodesis when factoring closure time for larger incision	
Reduced risk of infection and skin irritation compared with open approach	
Lower risk of neurovascular injury	
Faster learning curve than the subacromial arthroscopic approach	

Overall, this has become our preferred technique for arthroscopic LHBT tenodesis. Although we still perform an open subpectoral approach in young, high‐demand patients, we prefer this method in almost all other cases, especially when other concomitant surgical procedures are performed. In summary, high‐in‐the‐groove loop‐and‐tack tenodesis is a valuable addition to the armamentarium of any surgeon performing shoulder arthroscopy.

## DISCLOSURES

The authors (N.P., J.P.S.) declare the following financial interests/personal relationships which may be considered as potential competing interests: N.P. reports a relationship with DePuy Synthes Mitek Sports Medicine that includes consulting or advisory; reports a relationship with American Shoulder and Elbow Surgeons that includes committee member; reports a relationship with Orthopedics Journal that includes editorial board. J.P.S. reports a relationship with Arthrex that includes travel reimbursement; reports a relationship with DJO Surgical that includes travel reimbursement; reports a relationship with Stryker that includes travel reimbursement; reports a relationship with American Shoulder and Elbow Surgeons that includes abstract review committee member; reports a relationship with Arthroscopy Journal that includes editorial board. The other authors (M. Nicholes, M. Ngatuvai) declare that they have no known competing financial interests or personal relationships that could have appeared to influence the work reported in this article.

## FUNDING

This article was funded by AANA's Military Advanced Surgical Treatment (MAST) Program.
